# Disarming Ex-Combatants’ Minds: Toward Situated Reintegration Process in Post-conflict Colombia

**DOI:** 10.3389/fpsyg.2019.00073

**Published:** 2019-01-29

**Authors:** Sandra Baez, Hernando Santamaría-García, Agustín Ibáñez

**Affiliations:** ^1^Departamento de Psicología, Universidad de los Andes, Bogotá, Colombia; ^2^Centro de Memoria y Cognición, Intellectus-Hospital Universitario San Ignacio, Bogotá, Colombia; ^3^Physiology and Psychiatry, Pontificia Universidad Javeriana, Bogotá, Colombia; ^4^Laboratory of Experimental Psychology and Neuroscience, Institute of Cognitive and Translational Neuroscience, INECO Foundation, Favaloro University, Buenos Aires, Argentina; ^5^National Scientific and Technical Research Council, Buenos Aires, Argentina; ^6^Center for Social and Cognitive Neuroscience, School of Psychology, Universidad Adolfo Ibáñez, Santiago, Chile; ^7^Universidad Autónoma del Caribe, Barranquilla, Colombia; ^8^ARC Centre of Excellence in Cognition and its Disorders, Sydney, NSW, Australia

**Keywords:** collective violence, armed conflict, reintegration process, post-conflict Colombia, ex-combatants

## Abstract

Collective violence in the context of armed conflict impacts the economy, health systems, and social stability of affected countries. This is considered a complex phenomenon with interwoven biological, psychological, social, cultural, and political factors. However, most of the research on this topic still lacks suitable established integrative approaches to assess multilevel perspectives. Social, cognitive and affective mental processes (SCAMP) are critical factors that should be considered in multilevel approaches. In this article, we critically discuss some of the classically isolated approaches used in violence research, the absence of successful interventions for ex-combatants reintegration, and the specific neglect of SCAMP in these interventions. We present the case of post-conflict Colombia as a unique opportunity to study the different roots of collective violence, and we call for a more robust and situated approach to understanding of and intervention in this multifaceted phenomenon. In addition, we suggest a two-stage approach for addressing ex-combatants’ reintegration programs, which considers the situated nature of post-conflict scenarios and the urgent need for evidence-based interventions. This approach focuses on the comprehensive scientific assessment of specific factors involved in violence exposure and the subsequent design of successful interventions. The implementation of this approach will contribute to the effective reintegration of individuals who have been exposed to extreme violence for more than 50 years.

## Post-Conflict Colombia: Taking the Laboratory Into the Field

Collective violence within the context of armed conflicts is a scourge with great impacts on human society. Numerous civil wars and armed conflicts across the globe (e.g., Congo, Syria, Israel-Palestine, Colombia) exemplify the pervasive and ubiquitous nature of human violence (Muchembled, 2010). Collective violence in armed conflicts impacts the economy, health systems, and social stability of affected countries. Consequently, numerous efforts have been made to try to understand the determinants and consequences of this phenomenon. In this line, important research questions have been addressed by different approaches from the social sciences (Mike, 2018), biology (Raine, 2013) and, more recently, neuroscience (Poldrack et al., 2018). However, most of this research still lacks properly integrated and established approaches to assess multilevel (social, cultural, political, biological, and neurocognitive) perspectives. In this article, we question some of the classically isolated approaches that have been used for violence in armed conflicts research, the lack of evidence-based interventions that have been employed in the reintegration of ex-combatant’s or veterans, and the neglect of social, cognitive and affective mental processes (SCAMP) that has been seen in these interventions. We present the case of post-conflict Colombia as a unique scenario for studying the different roots of collective violence, and we call for a more robust, systematic and established approach for the understanding of and intervention in this multifaceted phenomenon. We suggest a two-stage approach (research on specific factors and subsequent development of evidence-based interventions) for addressing reintegration programs, which considers the established nature of post-conflict scenarios and the urgent need of evidence-based interventions.

The Colombian conflict is a great example of the inherent complexity of the sources, actors and effects of violence. Across more than 50 years of army conflict, Colombia has nearly 363,374 victims of violence. This includes 363,374 victims of threats, 22,915 victims of sexual offenses, 167,809 victims of enforced disappearance, 7,265,072 victims of forced displacement and 11,140 victims of anti-personnel mines (Amnesty International Report 2017/18, 2018). Different societal factors impact the Colombian armed conflict, including territorial conflicts, socioeconomic inequities, forced displacement and even cultural factors that normalize violent behaviors (Bohorquez et al., 2009; Reardon, 2018). Violence in Colombia has been exerted by drug cartels, Marxist–Leninist guerrillas (e.g., FARC and ELN), paramilitary armies, and national army forces, among other actors.

The government and the Revolutionary Armed Forces of Colombia (FARC) reached a peace agreement in 2016 to end their 52-year armed conflict. In 2017, the United Nations mission in Colombia verified that the FARC had handed over its weapons and demobilized. However, a minority of dissident guerrilla fighters rejected the terms of the peace agreement, have not disarmed, and continue to commit violent acts. Currently, civilians continue to face death threats and violence from National Liberation Army (ELN) guerrillas and paramilitary successor groups that emerged after a demobilization process that occurred a decade ago (Human Rights Watch World Report, 2018). Thus, post-conflict Colombia has represented a source of other types of violence rather than a peaceful scenario. This new sociopolitical climate has allowed for a renewed interest in exploring the origins, consequences, predictors, and resilience factors of violence, as well as the social-cognitive and psychological effects in ex-combatants, victims and civil populations (LeGrand et al., 2017; Flores and Vargas, 2018; Reardon, 2018). Moreover, it favors opportunities for developing integrative research and opens the door for designing new evidence-based and personalized interventions to propel social reintegration processes.

## The Lack of Current Reintegration Interventions in the Colombian Scenario

### The Neglect of Mental Health and SCAMP in the Ex-Combatants’ Reintegration

Ex-combatants usually display abnormal and exacerbated expressions of aggression and violent behavior (Betancourt et al., 2010; Hermenau et al., 2013; Kobach et al., 2015), which can persist even years after demobilization (Betancourt et al., 2010; Weierstall et al., 2012) (see Table 1 for a review of the available evidence). At the same time, combatants are exposed to numerous forms of extreme violence; they often witness, experience and perpetrate acts such as killing, torture, and rape. Exposure to violence, including organized violence, enhances the risk of mental disorders such as trauma-related illnesses, depression or substance abuse (Ikin et al., 2007; Odenwald et al., 2007a,b; Toomey et al., 2007; Ginzburg and Solomon, 2011; Maguen et al., 2011).

**Table 1:** Group-based experimental studies on long-term mental health symptoms or engagement in violent acts in ex-combatants or war veterans.

Study	Objective	Sample	Instruments	Procedure	Results
Meyer-Parlapanis et al., 2015	To investigate differences between male and female ex-combatants regarding their engagement in appetitive aggression	Four hundred and twenty-nine participants (412 males, 15 females) were recruited from a military and a rebel veteran’s organization in Bujumbura, Burundi. Taken from the larger male sample, 15 male former combatants were matched to the 15 female former combatants on the criteria of age, cumulative exposure to traumatic stressors, offense load and current post-traumatic stress disorder (PTSD) symptom severity. For a control population, 20 males and 20 females non-combatants were recruited as a random sample from the community.	(1) Post-traumatic Stress Diagnostic Scale, (2) PTSD Symptom Scale-Interview (PSS-I), (3) Appetitive Aggression Scale (AAS)	The interviews took place on the Université Lumière Bujumbura campus from July to September 2012 and at the Red Cross Burundi in Gatumba in January 2013. Five psychologists (3 males, 2 females) from the University of Konstanz, with the assistance of local interpreters (all male), conducted the assessments.	(1) The combatant group showed significantly higher levels of appetitive aggression than the non-combatant group (2) When matched by several war related variables and events, male and female combatants show statistically the same levels of appetitive aggression
Kobach et al., 2015	To replicate previous findings associating violent behavior with appetitive aggression, but not to post-traumatic stress in a sample of demobilized soldiers from Burundi.	Burundian ex-combatants, who were contacted through an official national veteran association. The final sample (*N* = 367) had an average age of 36 years (*SD* = 8.5, range: 19–62) and reported 7 years (*SD* = 3.0, range: 0–17) of formal education. On average, they had been recruited by the age of 19 years (*SD* = 4.2, range: 6–39), had spent 12 years (*SD* = 7.3, range: 0–35) in a (para) military group and had been demobilized 5.5 years (*SD* = 2.0, range: 0–14) before they conducted the interviews.	(1) Appetitive Aggression Scale (AAS), (2) PTSD Symptom Scale-Interview (PSS-I). (3) For exposure to violence a checklist was developed.	Interviews were conducted at the campus of the University Lumière in Bujumbura and took on average 1.5 h. Five clinical psychologists from the University of Konstanz, one clinical psychologist and six advanced students of clinical psychology from the University Lumière interviewed the participants.	(1) The number of perpetrated violent acts was the greatest predictor of appetitive behavior (2) The number of types of experienced traumatic events was the greatest predictor of PTSD
Augsburger et al., 2015	To investigate the impact of violent experiences during childhood, PTSD, and appetitive aggression on everyday violent behavior in Burundian females with varying participation in war.	One hundred and fifty-eight women in Burundi who had either been former combatants (*n* = 54), supporters of armed groups without involvement in fighting (*n* = 50), or civilians (serving as control group, *n* = 54).	(1) Post-traumatic Diagnostic Scale Checklist of self-committed violence, (2) Domestic and community violence check-list, (3) Appetitive Aggression Scale (AAS), (4) PTSD Symptom Scale-Interview (PSS-I)	Data collection was carried out in fall 2014 in Bujumbura, Burundi. Former armed group members were invited to the study with the help of a local contact person from an official national veteran association. Female civilians inhabiting the same neighborhoods as the former members of armed groups were invited to participate as controls. A team of experienced clinical psychologists from the University of Konstanz and trained local psychology students conducted the interviews.	(1) Appetitive aggression is greatest in the former combatant group, followed by the supporters group (2) Perpetrated violence across lifetime, exposure to traumatic events and PTSD were significantly higher in the former combatant group (3) Appetitive violence, PTSD symptoms and history of violent experiences during childhood predicted everyday violent behaviors
Nandi et al., 2015	To investigate the factors that are associated with the level of PTSD and appetitive aggression in former and still active combatants	Nine hundred and forty-eight male Burundian combatants of which 392 had been demobilized after war and 556 were still active as soldiers. Those still active in the military were preparing for deployment in the African Union Mission for Somalia. Participants in the demobilized group were contacted through an official national veteran association.	(1) Post-traumatic Stress Diagnostic Scale, (2) Composite International Diagnostic Interview (CIDI), (3) Symptom Scale Interview (PSS-I), (4) Appetitive Aggression Scale (AAS)	Interviews with the demobilized combatants were conducted at the campus of the Université Lumiére in Bujumbura, Burundi. Interviews with the active soldiers were mostly conducted at the military camp Mudubugu (Bubanza province, Burundi).	(1) The ex-combatants groups showed to have lived more traumatic experiences, childhood maltreatment, showed higher PTSD symptoms, and greater appetitive aggression. (2) There is higher risk for PTSD when number of traumatic stressors increases
Hecker et al., 2013	To investigate the impact of perpetrating violent acts on the perpetrator’s mental health.	Two hundred and four participants who belonged to a variety of armed groups and forces. In total, 43% were former members of the foreign armed group Forces Democratiques pour la Liberation du Rwanda and 57% of local armed groups including different Mai-Mai groups, Congres National pour la Defense du Peuple, or the Congolese Government Army. All participants were male reporting a mean age of 24.6 1 years.	(1) Sociodemographic information interview, (2) Checklist of war- and non-war-related potentially traumatic events from the checklist of the Post-traumatic Stress Diagnostic Scale, (3) the PTSD Symptom Scale-Interview, (4) the Appetitive Aggression Scale	In a semi-structured interview, respondents were questioned about appetitive aggression and PTSD as well as self-experienced violence and self-perpetrated violent offending. All interviews were conducted between March and May 20 II in Goma, in the province of North Kivu in the eastern Congo.	(1) Voluntary combatants perpetrated more violent acts and showed higher appetitive aggression. (2) Perpetrating violence was positively related to PTSD in forcibly recruited combatants, but not in voluntary combatants.
Elbogen et al., 2013	To identify whether self-reported violence problems that are associated with future violent behavior among a sample of Iraq and Afghanistan war veterans.	Three hundred participants (*n* = 150 dyads) of Iraq and Afghanistan war veterans and family/friends. The sample was predominately men (81%) with a median age of 39 years, 57% of the participants reported post high school education (57%).	(1) Structured interview for Diagnostic and Statistical Manual of Mental Disorders (SCID), (2) Traumatic life events questionnaire, (3) Combat Exposure Scale, (4) The Drug Abuse Screening Test (DAST), (5) The Alcohol Use Disorder, (6) Identification Test (AUDIT), (7) Conflict Tactics Scale, (8) MacArthur Community, (9) Violence Scale, (10) Violence question: “During the past 30 days, have you had trouble controlling violent behavior (that is, hitting someone)?”	The veterans completed baseline and follow-up interviews 3 years later on average, and family/friends provided collateral data on dependent measures at follow-up	(1) Combat exposure and PTSD symptoms predict severe violence (2) Combat exposure, PTSD, age, having witnessed parental violence in the past, and history of problems controlling violence predict other types of physical aggressions
Weierstall et al., 2012	To investigate whether the adaptive advantage in combatants who experience aggression to be appetitive also had very long-term protective and even beneficial effects on their mental health.	Fifty-one World War II male veterans that all experienced the Second World War as Germans. Participants had a mean age of 86.7 years (*SD* = 2.8; range = 81–95).	(1) PTSD Symptom Scale-Interview Version (PSS-I), (2) Hopkins Symptom Checklist (HSCL), (3) German Version of the Appetitive Aggression Scale	The convenience sample was recruited in different cities in the South-West of Germany by placing advertisements in the local newspapers and posting signs on bulletin boards in residential homes for the elderly. Data was collected between May and September 2010 using structured interviews. All interviews were carried out in participants’ homes and lasted for about 90 min.	(1) Ex-combatants who reported a higher appetitive aggression experienced less PTSD symptoms
Ginzburg and Solomon, 2011	To examine the chronological inter-relationships between post-traumatic stress reactions and somatization symptoms among combatants over a 20-year period	Two groups of Israeli male veterans: The first group consisted of 363 Israeli soldiers who fought in the Lebanon War and had been identified by military mental health personnel as suffering from combat stress reaction on the battlefield. The control group consisted of 301 soldiers who had participated in combat in the same units as those of the first group but were not identified as suffering from combat stress reaction. Groups were matched in age, education, military rank and assignment.	(1) Checklist of Negative life events in childhood, (2) Impact of Event Scale (IES), (3) Symptoms Checklist-90-R (SCL-90-R)–somatization subscale	Participants were assessed at four points of time: in 1983, 1984, 1985, and 2002. One, two, and three years following their participation in the war, participants were asked to report to the Headquarters of the Surgeon General to take part in this study. Participants filled out a battery of questionnaires in small groups. Twenty years after the war, data were collected at the veterans’ homes.	(1) Veterans diagnosed with combat stress reaction reported higher initial levels of intrusion and avoidance and a steeper decline in those symptoms over time in comparison to the control group. (2) Veterans diagnosed with combat stress reaction reported higher initial levels of somatization. (3) Over the years, stress reactions were positively associated with somatization symptoms for both groups
Ginzburg et al., 2010	(1) To follow-up the prevalence of comorbidity of PTSD, anxiety and depression; (2) to determine the chronological relations between these disorder; and (3) to examine whether PTSD comorbid with anxiety and depression is implicated in more impaired functioning than PTSD by itself.	Two groups of Israeli male veterans participated in this study. The first group consisted of 363 Israeli soldiers who fought in the Lebanon War and had been identified by military mental health personnel as suffering from combat stress reaction. The comparison group consisted of 301 soldiers who had participated in combat in the same units as those of the first group, but were not identified as suffering from combat stress reaction. The two groups were matched in age, education, military rank and assignment.	(1) The PTSD inventory, (2) Depression and anxiety subscales of the Symptoms Checklist-90 (SCL-90), (3) A 29-item self-report questionnaire, that assesses problems in psychosocial functioning.	War veterans were followed up 1, 2, and 20 years after their participation in the 1982 Lebanon War. Participants with missing data on either Time 2 or Time 3 assessment were included in the sample.	(1) At each point of assessment, rates of triple comorbidity (PTSD, anxiety and depression) were higher than rates of PTSD, either by itself, or comorbid with depression or anxiety. (2) PTSD predicted depression, anxiety, and comorbid disorders, but not vice versa. (3) At time 1 and 2, triple comorbidity was associated with more impaired functioning than PTSD alone. (3) Triple comorbidity at Time 2 was associated with more impaired functioning than double comorbidity.
Maguen et al., 2011	To examine factors that are associated with increased suicidal ideation in returning Iraq War veterans.	Cases included 2,854 Operation Iraqi Freedom soldiers who presented for their routine post-deployment screening from November 2005 to June 2006. Soldiers had a mean age of 28 years (*SD* = 6; range = 19–52).	(1) Soldiers reported demographic and military service variables, (2) Questions assessing risk factors for suicidal ideation: (a) “Have you ever attempted to kill yourself?” (b) “Do you have relatives who have attempted suicide?” (c) “Have you ever been on any medication for emotional problems?” and (d) “Have you received mental health or alcohol counseling in the past?” (3) Questions to assess combat exposure: (a) “During combat operations did you become wounded or injured?” (b) “During combat operations did you see the bodies of dead soldiers or civilians?” (c) “During combat operations did you personally witness anyone being killed?” and (d) “During combat operations did you kill others in combat (or have reason to believe that others were killed as a result of your actions)?” (5) Primary Care PTSD Screen (PC-PTSD), (6) Patient Health Questionnaire-8 (PHQ-8), (7) Alcohol Use Disorder Identification Test (AUDIT), (8) Suicidal ideation at post-deployment screening: (a) “Over the last 2 weeks, how often have you had thoughts that you would be better off dead, or of hurting yourself in some way?” and (b) “Do you feel like hurting yourself at this time?”	Data were derived from a post-deployment screening database at a large Army medical facility. All cases that returned from Operation Iraqi Freedom deployments were eligible for inclusion. The program provides a general health assessment, including mental health screening. In this study, soldiers completed a set of screening measures and self-reported demographics and deployment-related information. Medical personnel for injury prevention, smoking cessation, or other reported physical or mental health concerns subsequently saw soldiers.	(1) Overall, 2.8% of soldiers reported suicidal ideation. (2) Post-deployment depression symptoms were associated with suicidal thoughts, while post-deployment PTSD symptoms were associated with current desire for self-harm. (3) Post-deployment depression and PTSD symptoms mediated the association between killing in combat and suicidal thinking, while post-deployment PTSD symptoms mediated the association between killing in combat and desire for self harm.
Maguen et al., 2010	To examine the relationship between killing and mental health in returning Iraq War veterans.	Participants included 2,797 Operation Iraqi Freedom soldiers who presented for their post-deployment screening from November 2005 to June 2006. Participants had a mean age of 28 years (*SD* = 6; range = 18–52).	(1) Soldiers reported age, gender, race/ethnicity, educational status, relationship status, (2) they responded to three questions to assess level of combat exposure: (a) During combat operations did you become wounded or injured? (b) During combat operations, did you see the bodies of dead soldiers or civilians? (c) During combat operations, did you personally witness anyone being killed? (3) Soldiers responded to the following question to assess direct and indirect killing experiences, “During combat operations did you kill others in combat (or have reason to believe that others were killed as a result of your actions)?” (4) Primary Care PTSD Screen, (5) Patient Health Questionnaire (PHQ-9), (6) Alcohol Use Disorder Identification Test (AUDIT), (7) Dimensions of Anger (DAR).	Data were derived from a post-deployment screening database at a large Army medical facility. All participants who returned from Operation Iraqi Freedom deployments were eligible for participation. The program provides a general health assessment, including mental health screening. In this study, soldiers completed a set of screening measures and self-reported demographics and deployment-related information. Medical personnel for injury prevention, smoking cessation, or other reported physical or mental health concerns subsequently saw soldiers.	(1) After controlling for combat exposure, killing was a significant predictor of PTSD symptoms, alcohol abuse, anger, and relationship problems. (2) Military personnel returning from modern deployments are at risk of adverse mental health conditions and related psychosocial functioning related to killing in war.
Milliken et al., 2007	To measure the mental health needs among soldiers returning from Iraq and the association of screening with mental health care utilization.	Population-based, longitudinal descriptive study of the initial large cohort of 88,235 US soldiers returning from Iraq who completed both a Post-Deployment Health Assessment and a Post-Deployment Health Re-Assessment.	(1) Post-Deployment Health Reassessment form, (2) A 2-item depression instrument from the Patient Health Questionnaire (PHQ), (3) The Primary Care 4-item post-traumatic stress disorder screen (PC-PTSD), (4) A question on suicidal ideation from the PHQ, (5) A question on interpersonal aggressive ideation, asking if the soldier is “having thoughts or concerns that you might hurt or lose control with someone,” (6) Interpersonal conflict was measured with one question that asks if the soldier is “having thoughts or concerns that you may have serious conflicts with your spouse, family members, or close friends.”	Between June 1, 2005, and December 31, 2006, Army soldiers and Marines completed Post-Deployment Health Reassessment forms. The soldiers completed the form a median of 6 months after return home. Active component soldiers were followed up for 90 days after completion to determine their health care use.	(1) Based on a combined screening, clinicians identified 20.3% of active and 42.4% of reserve component soldiers as requiring mental health treatment. (2) Concerns about interpersonal conflict increased fourfold. (3) Soldiers frequently reported alcohol concerns, yet very few were referred to alcohol treatment.
Toomey et al., 2007	To assess the prevalence of war-era onset mental disorders in United States veterans deployed to the Gulf War and in non-veterans 10 years after the war	One thousand and sixty-one deployed veterans and 2,883 non-deployed veterans. Both groups were 78% male. The deployed group (mean age 38.9 years), were nearly 2 years younger than the non-deployed group (40.7 years).	(1) Clinician Administered PTSD Scale (CAPS), (2) Composite International Diagnostic Inter- Composite International Diagnostic Interview (CIDI), (3) The PTSD Checklist, (4) The Beck Depression Inventory II (BDI-II), (5) The Beck Anxiety Inventory (BAI), (6) The 36-item Short Form Health Survey (SF-36), (7) The Quality of Life Inventory (QoLI), (8) The Combat Exposure Scale (CES)	The United States Department of Defense’s Defense Manpower Data Center identified the entire cohort of 693,826 deployed veterans and approximately half of the non-deployed veterans who were in military service between September 1990 and May 1991. For the examination phase of the study, a list of potential participants was created by random selection. Potential participants were assigned to the participating Veterans Affairs Medical Centre closest to their home.	(1) Gulf War-era onset mental disorders were more prevalent in deployed veterans compared with non-deployed veterans. (2) The prevalence of depression and anxiety declined 10 years later in both groups, but remained higher in the deployed group, who also reported more symptoms and a lower quality of life. (3) Remission of depression was be related to the presence of comorbid psychiatric disorders and level of education. (4) Remission of anxiety was related to treatment with medication.
Ikin et al., 2007	To investigate the association between war service, anxiety, PTSD and depression in Australia’s surviving male Korean War veterans.	The veterans group comprised 7,612 male Korean War veterans, representing all of those considered to be still alive and residing in Australia. Female veterans constituted only 0.3% of the original deployment, and were 0.3% of the original deployment, and were excluded from the study because of their ex- excluded from the study because of their extremely small numbers. The comparison group was composed by 1,150 members of the community.	(1) The 14-item Hospital Anxiety and Depression (HAD), (2) Post-traumatic Stress Disorder Checklist, (3) Combat Exposure Scale (CES).	The sample was recruited by means of a postal invitation, with two further mailings postal invitation to non-responders. Demographic and health information and some Korean War service characteristics were collected by means of a self-report postal questionnaire. Data on additional Korean War service characteristics were obtained from Department of Veterans’ Affairs’ records.	(1) PTSD, anxiety and depression were more prevalent in veterans than in the comparison group. (2) These disorders were strongly associated with heavy combat and low rank.
Odenwald et al., 2007b	To develop and validate of a screening tool for PTSD in Somali language with a sample of ex-combatants.	One hundred and thirty-five participants involved in three different sections of the Somaliland armed forces: army, police, and custodian corps^2^. All participants were former members of the Somali National Movement. 133 were men and two were women. Their ages ranged from 19 to 70 years.	(1) Modified version of the Post-traumatic Stress Diagnostic Scale (PDS), (2) Composite International Diagnostic Interview for the DSM-IV (CIDI)	We adapted the Post-traumatic Diagnostic Scale (PDS) to reflect linguistic and cultural differences within the Somali community so that local interviewers could be trained to administer the scale.	(1) The screening instrument is a reliable and valid method to detect PTSD among Somali ex-combatants. (2) Ex-combatants with a positive screening outcome reported more anxiety and depression-related symptoms, more psychotic symptoms and more minor physical problems.
Odenwald et al., 2007a	To provide information about drug abuse among Somali active security personnel and militia with an emphasis on regional differences in relation to the lack of central governmental control.	In total, 8,124 militiamen and security staff were interviewed. Of them, 4,070 belonged to regional authorities and 2,290 to warlord factions, 1,090 were members of freelance and clan-based militia, 481 of Sharia court militias, and 78 members of business militias.	(1) A questionnaire designed as a standardized instrument to asses: basic socio-demographic information, self-reported khat use, and how respondents perceived the use of khat, cannabis (which includes both hashish and marijuana), psychoactive tablets (e.g., benzodiazepines), alcohol, solvents, and hemp seeds in their units.	Interviews were conducted between August and December 2003. The interviewers went directly into a compound used by the respective militia or units to conduct the individual interviews in a place that provided as much privacy as possible, e.g., in a separate room.	(1) In total, 36.4% of respondents reported khat use in the week before the interview. (2) The most frequent form of drug use is khat chewing, followed by smoking cannabis, ingesting psychoactive tablets, drinking alcohol, inhaling solvents and eating hemp seeds. (3) Perceived use of khat differs little between northern and southern Somalia, but perceived use of other drugs reaches alarmingly high levels in some regions of the south.
Boscarino, 2006	To examine all-cause and cause-specific mortality among a national random sample of United States Army veterans with and without PTSD after military service.	Men who served in the United States Army during the Vietnam War. The cohort was identified through a random sample of 48,513 service records selected from the nearly five million records on file at the National Personnel Records Center. Of these, 18,581 veterans met the criteria for study eligibility, which were chosen to increase comparability between men who served in Vietnam and men who served elsewhere.	(1) The following variables were measured at interview: race, Army volunteer status, Army entry age, Army discharge status, Army illicit drug use, intelligence, and age at interview, pack-years of cigarette smoking. (2) Diagnostic Interview Scheduled Version III (DIS-III) PTSD Scale	We used Cox proportional hazards regressions to examine the causes of death among 15,288 male United States Army veterans 16 years after completion of a telephone survey, approximately 30 years after their military service. These men were included in a national random sample of veterans from the Vietnam War Era.	(1) Adjusted postwar mortality for all-cause, cardiovascular, cancer, and external causes of death (including motor vehicle accidents, accidental poisonings, suicides, homicides, and injuries of undetermined intent) was associated with PTSD among Vietnam Theater veterans. (2) For Vietnam Era veterans with no Vietnam service, PTSD was associated with all-cause mortality.
Benyamini and Solomon, 2005	To examine the association of initial combat stress reaction, chronic PTSD and cumulative life stress on physical health 20 years after the 1982 war with Lebanon, in a sample of Israeli veterans of the war.	The sample included 504 male veterans who took part in active combat in the Lebanon War in 1982. Two groups participated in the study: The combat stress reaction group (*n* = 286) and (b) The combat control group with no CSR (*n* = 218). Groups were paired by socio-demographic characteristics (age, education, and military rank and assignment).	(1) The post-traumatic stress disorder inventory, (2) A self-report questionnaire was designed to evaluate physical health, (3) Cumulative life stress was assessed with a checklist of 20 life events in several domains.	Twenty years following the war, participants were asked to rate their general physical health status, report health complaints and risk behaviors, and were screened for PTSD. Those who agreed to participate in the study were offered to meet in their homes or in another preferred location to complete the questionnaire.	(1) PTSD was associated with general self-rated health, chronic diseases and physical symptoms, and greater engagement in risk behaviors. (2) Combat stress reaction and PTSD were related to greater cumulative life stress since the war. (3) PTSD suppressed the effects of additional life stress (negative life events had a weaker effect on health among participants with PTSD).
Ismail et al., 2002	To examine the prevalence of psychiatric disorders in veterans of the Gulf war with or without unexplained physical disability and in similarly disabled veterans who had not been deployed to the Gulf war (non-Gulf veterans).	Phase 1 consisted of three randomly selected samples of Gulf veterans, veterans of the 1992–1997 Bosnia peacekeeping mission, and United Kingdom military personnel not deployed to the Gulf war (Era veterans) who had completed a postal health questionnaire. Phase 2 consisted of randomly selected subsamples from phase 1 of Gulf veterans who reported physical disability (*n* = 111) or who did not report disability (*n* = 98) and of Bosnia (*n* = 54) and Era (*n* = 79) veterans who reported physical disability.	(1) SF-36 physical functioning subscale, (2) The World Health Organization’s schedule of clinical assessment in neuropsychiatry, a semi-structured interview.	It was a two-phase study design. Phase 1 was a population-based survey using a postal health questionnaire to compare self-reported health indices in three randomly selected cohorts of the United Kingdom armed forces. In phase 2, authors compared Gulf veterans who screened positive at phase 1 for physical disability with Gulf veterans who screened negative and with Bosnia and Era veterans who screened positive.	(1) Only 24% of the disabled Gulf veterans had a formal psychiatric disorder (depression, anxiety, or alcohol related disorder). (2) The prevalence of psychiatric disorders in non-disabled Gulf veterans was 12%. (3) Disability and psychiatric disorders were weakly associated in the Gulf group. (4) The prevalence of psychiatric disorders was similar in disabled non-Gulf veterans and disabled Gulf veterans. (5) All groups had rates for post-traumatic stress disorder of between 1 and 3%.
McFall et al., 1999	To test the hypothesis that violence among Vietnam veterans seeking specialized inpatient care for PTSD is more prevalent compared with (a) psychiatric inpatients without PTSD and (b) a community sample of Vietnam veterans with PTSD who have not undergone inpatient treatment.	Participants diagnosed with PTSD were 228 male Vietnam combat veterans. Their average age was 46.32 (*SD* = 2.49) years. Over 80% of participants had completed the equivalent of 12 years of education (*M* = 13.6, *SD* = 2.17). A sample of (a) 64 male psychiatric patients without PTSD and (b) 273 male Vietnam veterans with PTSD who have not undergone inpatient treatment, served as comparison groups in this study.	(1) Mississippi Scale for Combat-Related PTSD, (2) Global Assessment of Functioning (GAF), (3) Conflict Tactic Scale (CTS), (4) 10-item RCES, (5) War Stress Intake Questionnaire (WSIQ)	Violent acts within the PTSD and non-PTSD psychiatry inpatient samples were measured by patient self-report on a questionnaire administered during hospitalization. Nine items adapted from the CTS assessed violence in the sample of community residents with PTSD.	(1) Before seeking hospitalization, PTSD from the inpatient group were more violent than the PTSD group that did not seek hospitalization. (2) Inpatients with PTSD were categorized as highly violent in contrast to other psychiatric inpatients that did not have PTSD.


Despite this evidence, disarming and reintegrating *the mind* of ex-combatants is not currently considered a critical component of reintegration. Mental health and SCAMP have not been considered to be core topics for Colombian disarmament, demobilization, and reintegration (DDR) programs. Indeed, the effects of violence- and conflict-exposure on the mental health of ex-combatants have not been investigated in a rigorous and comprehensive manner. In addition, social cognition abilities have been proposed as important variables in relation to violent profiles (Bennette et al., 2005; Harenski et al., 2010; Jusyte and Schonenberg, 2017). In particular, social cognition skills allow individuals to encode information, interpret and predict the consequences of a particular action, and determine an appropriate response. It has been suggested (Bennette et al., 2005) that certain ways of processing social information help to protect the individual from personal, social, environmental, or situational pressures toward violent behavior. In addition, some studies have shown that, compared to non-violent offenders, violent offenders are significantly poorer on facial-affect recognition (Hoaken et al., 2007) and exhibit a deficit in the categorization of fearful expressions (Jusyte and Schonenberg, 2017). This deficit is associated with self-reported aggression and psychopathy levels (Jusyte and Schonenberg, 2017). In addition, criminal psychopaths show abnormal processing of moral scenarios (Harenski et al., 2010). They showed a negative association between moral violation severity ratings and posterior temporal activity that was not present in non-psychopaths.

### Evidence on Neuroscience of Group Conflicts

Previous evidence has revealed a relationship between emotions and attitudes involved in intergroup conflicts. For instance, interpersonal-disgust sensitivity predicts negative attitudes toward immigrants, foreigners, and socially deviant groups (Hodson and Costello, 2007). This effect is mediated by ideological orientations (social dominance orientation, right-wing authoritarianism) and dehumanizing perceptions of the out-group. Moreover, anger influences automatic evaluations of out-groups because of its functional relevance to intergroup conflict and competition (DeSteno et al., 2004). Other negative emotions less relevant to intergroup relations (e.g., sadness) result in non-automatic intergroup bias (DeSteno et al., 2004).

In this line, cognitive neuroscience has investigated the neural basis of prejudice and stereotyping in an effort to identify the processes through which these biases influence behavior. Neuroimaging studies have shown that the amygdala is associated with race-related processing (Lieberman et al., 2005) and that the amount of amygdala activity correlates with race-related prejudice (Cunningham et al., 2004). In this context, participants with greater implicit racial bias showed greater recruitment of brain regions supporting cognitive conflict and control (dorsal anterior cingulate cortex, dorsolateral prefrontal cortex, ventrolateral prefrontal cortex) (Richeson et al., 2003). This implicit racial bias modulates brain activity at very early time windows (Ibanez et al., 2009, 2010). These findings have allowed elucidating basic mechanisms of the social brain while advancing the understanding of intergroup bias in social behavior.

Additional evidence on neuroscience of group conflicts has approached to the understanding of neural markers of violent attitudes or behaviors. For instance, it has been found that youngsters who grow up in a climate of long-standing intergroup conflict inhibit the brain’s automatic response to the pain of out-group members (Levy et al., 2016). Tighter brain-to-brain synchrony among group members in the Arab-Palestinian minority enhanced the neural in-group bias (Levy et al., 2016). In the same line, a fMRI study in Arab, Israeli and control individuals showed that attitudes toward the out-group are predicted by activity in the precuneus. Other brain regions that were involved in reasoning about emotionally laden information did not show this pattern (Bruneau and Saxe, 2010). Besides, it has been suggested (Molenberghs et al., 2016) that people show greater moral sensitivity for in-group versus out-group victims, but only when the perpetrator is from the out-group. This effect correlates with greater activity in the orbitofrontal cortex for in-group victims when out-group individuals harmed them. It has been also reported that outcomes of social group competition can directly affect primary reward-processing neural systems (Cikara et al., 2011), which has implications for inter-group harm. Specifically, the ventral striatum activity, associated with subjective pleasure, also correlated with self-reported likelihood of aggressiveness against out-group members.

Together, available evidence suggests that social cognition abilities are relevant variables related to violent behaviors. Antecedents also highlight the importance of understanding how social factors shape violent behavior and the specific neural markers associated with violence in intergroup conflicts, and also emphasize the need to characterize the social cognition abilities of ex-combatants. Additionally, the findings of these studies underline the importance of including the stimulation/rehabilitation of social cognition processes in reintegration programs, as this is a crucial step to enhance the ability of ex-combatants to positively interact with social challenges in post-conflict scenarios.

### Limitations of Current Interventions

To date, the effects of long-term exposure to violence and its impact on mental health are poorly understood. Some studies suggest an increased risk of developing post-traumatic stress disorder, depression or substance abuse (e.g., Ikin et al., 2007; Odenwald et al., 2007a,b; Toomey et al., 2007; Ginzburg and Solomon, 2011; Maguen et al., 2011, see Table 1 for a review of the available evidence); however, other aspects of mental health, such as the presence of axis 1 and 2 psychiatric symptoms/disorders and SCAMP, have not been systematically assessed in ex-combatants. Additionally, two major limitations of current research approaches is the use of problematic instruments for assessing mental health, such as self-report measures, and the small samples sizes. The neglect of SCAMP can directly impact the degree to which reintegration is successful after conflict. Thus, the characterization of the psychopathologic, psychiatric, and social-cognitive profiles of ex-combatants is crucial for the comprehensive implementation and evaluation of such programs.

Efforts to demobilize and disarm armed factions and to reintegrate ex-combatants into civilian life are critical for peace building processes (Humphreys and Weinstein, 2007), as these factors are essential in preventing the recurrence of conflict. However, in Colombia, most DDR interventions have been designed with consideration of political, security, occupational and socioeconomic dimensions, while neglecting mental health or social-cognitive disorders of ex-combatants. Despite the remarkable importance of such programs, most of the available interventions have crucial limitations that have not been addressed, including: (a) reduced scientific assessment of ex-combatants’ mental health and SCAMP (as well as the concomitant scarcity of interventions focused on this aspect), (b) absent evidence regarding contextual and individual factors that explain whether individuals can successfully reintegrate after conflict, and (c) lack of rigorous assessment of intervention effectiveness and long-term effect.

Through the comprehensive characterization of the mental profiles of ex-combatants, it would be possible to design better interventions for the successful social reintegration of these individuals with potential long-terms effects. Only a few studies have attempted to provide evidence for the effectiveness of mental health interventions in ex-combatants (e.g., Hermenau et al., 2013; Trujillo et al., 2017, see Table 2 for a review of available evidence). Overall, these studies showed that specific interventions can reduce posttraumatic stress symptoms and aggressive attitudes and can enhance emotion recognition abilities. However, data on the long-term effects and the generalizability of these interventions are lacking.

**Table 2:** Group-based experimental studies on social-cognitive or mental health intervention in ex-combatants or war veterans.

Study	Sample	Procedure	Intervention program	Results
Trujillo et al., 2017	Thirty-one ex-combatants from Colombian illegal groups, 29 men, aged between 27 and 57 (*M* = 37.16, *SD* = 8.30) with an average education of 10.23 years (*SD* = 3.03).	The sample was divided into two groups. The first group (*n* = 16) received the Social Cognitive Training Intervention (SCT). The second group, followed the Conventional Reintegration Program (*n* = 15).	The Social Cognitive Training (SCT) Intervention. It was a low-intensity, brief (45 min, 12 sessions) Individual intervention aimed to improve social skills, theory of mind and emotional processing.	The SCT (1) significantly improved the recognition of neutral faces (2) reduced aggressive attitudes, (3) reduced the aggressive behavior triggers.
Kearney et al., 2016	Fifty-five veterans with Gulf War Illness, defined as deployment to the Gulf War theater of operations between August 1990 and August 1991 and self-report of at least two of the following symptoms: (1) fatigue that limits usual activity; (2) musculoskeletal pain involving two or more regions of the body; (3) cognitive symptoms (memory, concentration, or attention difficulties)	War Veterans were randomly assigned to treatment as usual plus Mindfulness-Based Stress Reduction (MBSR) or treatment as usual only. Pain, fatigue, and cognitive failures were assessed at baseline, post-MBSR and at 6-month follow-up. Secondary outcomes included symptoms of post-traumatic stress disorder and depression.	Mindfulness-Based Stress Reduction was delivered in 8 weekly, 2.5 h sessions plus a single 7-h weekend session.	Veterans randomized to MBSR plus treatment as usual reported: (1) Greater reductions in pain, fatigue and cognitive failures, (2) greater decline in depressive symptoms and (3) greater reductions in post-traumatic stress symptoms.
Yaganeh et al., 2014	Thirty war veterans suffering from post-traumatic stress disorder (PTSD). Patients between 40 and 60 years, having under constant medication, attendance in neurofeedback treatment, and having the least ability in reading and writing.	Patients with PTSD were randomly assigned to neurofeedback training (*n* = 15) and control (*n* = 15) groups. Data were collected by PTSD checklist. Groups were evaluated for intensity of symptoms at the beginning and end of the study.	Neurofeedback training was implemented for 20 sessions, 3 days per week. All subjects were trained α/θ protocol for 45 min.	The neurofeedback training significantly reduced the PTSD symptoms as measured by the PTSD checklist.
Hermenau et al., 2013	Fifty-eight male former combatants and child soldiers who belonged to a wide range of militia and self-defense groups, including the Democratic Forces for the Liberation of Rwanda, the National Congress of the People and several local Mai-Mai militia groups. Age (*M* = 19.00, *SD* = 2.20), education (*M* = 6.13, *SD* = 3.98).	Out the sample of 58 participants at the baseline assessment, 38 participants were present at the time of the pretest and matched into 19 pairs of ex-combatants. They were randomly assigned to the intervention or the control group (did not receive intervention). Matching criteria were symptoms of post-traumatic stress (PTSD) and appetitive aggression.	The Narrative Exposure Therapy for Forensic Offender Rehabilitation (FORNET) aims to reduce both PTSD symptoms and appetitive aggression by recalling the experiences through narrative exposure. It helps the ex-combatant to anchor not only fearful and traumatic experiences, but also positive feelings that might have been linked to various forms of aggressive behavior in the past. The role change from a combatant to a civilian is specifically addressed and reinforced.	(1) The FORNET reduced post-traumatic stress. (2) Ex-combatants treated with FORNET were more able to find closure with their past. (3) Appetitive aggression decreased in both groups.
Ready et al., 2008	The study included 93 (91%) Vietnam veterans, 4 (4%) Gulf War veterans, 2 (2%) Iraq War veterans, 2 Korean War veteran (2%) and 1 (1%) veteran who came under enemy fire and was nearly killed in a natural disaster during a peace-keeping mission in the 1990s. All were male except one Vietnam veteran who was a nurse. The average age at the start of treatment was 54 (*SD* = 6) and the ages ranged from 33 to 78.	All patients in this field test were referred for specialized PTSD treatment. All participants received the group-based exposure therapy. Clinician-administered and self-report measures of PTSD were acquired prior to treatment, post-treatment, and 6-month post-treatment	Group-based exposure therapy is a manualized outpatient program. Nine to 11 patients attended 3 h of group therapy per day twice weekly for 16–18 weeks. Group-based exposure therapy is comprised of three phases: a didactic training and group-building phase, an exposure therapy phase, and a grief/guilt and relapse prevention phase.	The group-based exposure therapy produced a significant reduction of PTSD symptoms (re-experiencing, avoidance numbing, physiological reactivity). 81% of patients with baseline and follow-up data had a clinically significant improvement when comparing baseline to 6-month follow-up assessments.


## Toward a Two-Stage Approach for Reintegration Interventions

### First Stage: Understanding the Complexity of Collective Violence in Colombian Conflict

Although there is a large body of literature on different aspects of violence that spans across centuries, new approaches that propel more integrated views combining social, cultural, political, biological and neuroscientific perspectives are still lacking. Moreover, general theoretical models of collective violence in armed conflicts should be supported with an established, regional approach that considers the intrinsic local factors involved in the conflict. The first stage of the proposed approach involves the development of an established model that considers the contextual conditions in which violence has emerged and assesses the interaction between interpersonal and intrapersonal factors that could promote violent behaviors. Thus, before designing reintegration programs, it is crucial to understand the specific interplay between biological, psychological, social, political, and cultural factors involved in specific violent behaviors.

Violent behaviors to be studied by this approach should include different domains, according to the reasons to be involved in direct violence. These include: (a) consequentialist violence: violence committed by utilitarian reasons following the principle of the end justify means (Baez et al., 2017); (b) appetitive violence: primary pleasure or enjoyment in executing illegal violent acts (Elbert et al., 2018); (c) retaliatory violence: violent acts as a result of revenge or retaliation (Chester and DeWall, 2016; Thrasher and Handfield, 2018); and (d) impulsive violence: aggressive or violent acts as a consequence of impulsive, uncontrolled anger episodes, associated to poor behavioral control mechanisms (Elbert et al., 2018).

Interdisciplinary efforts are urgently needed to build novel strategies for violence research that incorporate inclusive approaches regarding violence origins, individual differences in SCAMP and mental health, and other factors that perpetuate violence and their consequences. Similarly, it is crucial to assess the background, including the cultural, social, and political context where reintegration interventions will be implemented. Specifically, new research approaches should consider the assessment of both contextual (social, cultural, and political) and individual or person-centered (e.g., mental disorders symptoms, personality traits, cognitive and social cognitive skills, physical health, quality of life aspects) factors that could be associated with different domains of violence. For instance, contextual factors include the presence of threatening social experiences (e.g., stigmatization, discrimination or exclusion, antecedents of intrafamilial abuse, the accessing to social, educational and occupational resources, the presence of political situations that impel social conflict (e.g., censured expressions of political ideas and neglected group participation, the level of membership identification with the armed group, the presence of beliefs and attitudes that normalize and idealize the actions of armed groups and the presence of beliefs and attitudes toward the compliance for juridical regulations.) Contextual assessment should include experiences and factors present at different stages of subjects’ life: before the subjects entered to the armed group, during the time they were part of the armed group, and after they leaved the armed group and accepted the reintegration processes.

The assessment of person-centered factors should include mental disorders symptoms, personality traits, basic cognitive abilities, social cognition skills and quality of life aspects. The last one involves the environment adaptation skills, social and emotional skills (skills to regulate emotional responses, resources to integrate social groups, presence of intimate relationships), determinants of psychical integrity and health (resources to satisfy basic health needs, presence of symptoms of physical alterations); and the self-projection skills.

Research approaches including a comprehensive assessment of both contextual and person-centered factors associated with different violence dimensions in ex-combatants, would contribute to the understanding of the specific interplay multidimensional factors involved in specific violent behaviors. Additionally, personalized assessments should identify both protective and risk factors for successful reintegration. Finally, more studies that anticipate the factors that may challenge successful reintegration and social participation processes are of critical relevance. This information is crucial for designing specific interventions within reintegration programs.

### Second Stage: Designing and Assessing Reintegration Interventions

The second stage involves the design of evidence-based interventions grounded in the knowledge accumulated during the first stage. Given the scarcity of interventions designed specifically for ex-combatants, successful interventions employed in other populations may be considered examples for future designs.

For instance, some research in healthy non-offender populations (Leiberg et al., 2011; Klimecki et al., 2013) has shown that short-term mental training using meditation-based techniques increases prosocial behavior and positive affect toward people who were not specifically targeted during training. Contemplative mental training increases perceived social connectedness, reduces the detrimental effects of loneliness (Kok and Singer, 2017) and improves interoceptive accuracy and emotional awareness (Bornemann and Singer, 2017). In addition, mental training interventions for different cognitive and social skills can induce specific changes in brain morphology (Valk et al., 2017). These brain changes are correlated with training-induced behavioral improvements in domain-specific measures of attention, compassion, and perspective-taking. Similarly, evidence-based social interventions exist for patients with psychiatric disorders such as autism or schizophrenia (Kandalaft et al., 2013; Lindenmayer et al., 2013; Roberts et al., 2014). These interventions also have been shown to be useful in increasing social-cognitive abilities and social functioning. Preliminary studies (Mullins, 2010) have also been adapted into rehabilitation programs. Though originally designed for ordinary offenders, these approaches are being considered for the disengagement and deradicalization of Islamist terrorists. These types of interventions are focused on generating behavioral and belief changes. To date, there is no evidence for the long-term effectiveness of these interventions, and they have not been rigorously employed in ex-combatants from armed groups. Thus, empirical support for their effectiveness is urgently needed, as they have potential applications that may be useful in ex-combatants.

New technologies and advances in data analysis should also be considered for designing interventions. For instance, recent research has revealed the utility of machine learning and data-mining methods to develop statistical models that aim to predict the risk for reoffense in violent criminals. These methods have the capacity to empirically discover patterns in the data and to construct suitably complex decision boundaries by using conventional predictors and a large sample size (Berk and Bleich, 2013). In this regard, recent studies have shown that risk assessments based on machine learning forecasts can improve parole release decisions (Berk, 2017) and might be able to provide timely and useful risk assessments for domestic violence (Berk et al., 2016). Additionally, these methods have been successfully used in forensic psychiatry to evaluate the risk for reoffense in mentally disordered individuals (Pflueger et al., 2015). In ex-combatants, machine-learning methods have been applied to regress specific traumatic events on appetitive aggression levels and post-traumatic stress profiles (Kobach et al., 2014). This study showed that the number of perpetrated violent acts was the best predictor of appetitive aggression, while traumatic events experienced was the best predictor of post-traumatic stress. Considering the available evidence from ordinary offenders and ex-combatants, machine learning and data-mining methods may be useful to evaluate the risk for reoffense, as well as to identify specific mental health profiles in ex-combatants and to design more personalized interventions.

In addition, insights from neuroscience could contribute to the understanding and (potentially) the prediction of violence and other forms of antisocial behavior present in ex-combatants. Cognitive neuroscience may contribute to the understanding of the neural basis of basic cognitive and social functions associated to violent behaviors. Besides, basic neuroscience could provide insights on aspects of brain function, structure, chemistry, or connectivity related to violence dimensions (Poldrack et al., 2018). In addition, some studies (Blair, 2013; Viding et al., 2014) have suggested the potential utility of neuroscience’s methods for predicting future violent behavior. Other promising results (Doehrmann et al., 2013; Goldstein-Piekarski et al., 2016) suggest that neuroimaging measures can be incorporated as modeling tools to assess the predictive validity of treatment outcomes. The utility of neuroscience’s methods for predicting future violent behavior offers the potential to identify critical neurocognitive mechanisms that distinguish between individuals who might benefit from treatment or preventive measures.

Specifically, our approach proposes the designing of long-term (at least 9 months) interventions that integrates individual, group and community strategies, matching aims with individual needs. Regarding individual interventions, it is worth to highlight that personalized interventions considering the individualized prescription of specific therapeutics (Schork, 2015) have been proposed as medical treatments. A similar approach could be considered in designing reintegration and rehabilitation interventions for ex-combatants. Individual differences in experience, ranging from intergenerational violence, sociopathy, psychopathy, and psychopathological (and even cognitive) profiles, are distributed across ex-combatant populations. Taking individual variability into account is essential for achieving successful reintegration into civilian life. In addition, these interventions should consider the context in which exposure to violence occurred and the socioeconomic and cultural characteristics of the geographic zone where reintegration will take place.

For instance, the proposed approach includes the adaptation of successful mental training interventions (Leiberg et al., 2011; Klimecki et al., 2013; Bornemann and Singer, 2017; Valk et al., 2017) for different cognitive and social skills, according to the ex-combatant’s needs. These interventions may be implemented in small-group sessions and should consider different social cognition domains (i.e., emotion processing, theory of mind, empathy, and moral reasoning). For instance, following previous protocols implemented in healthy non-offender participants (Valk et al., 2017), group interventions may include various daily mental exercises and weekly instructed sessions. Specifically, training protocols would comprise meditation-based techniques and dyadic interpersonal exercises (Valk et al., 2017). At the beginning of each of training module, participants would be part of a retreat (intensive training phase). There, they would be introduced to the core exercises and ideas of the module and would learn to integrate the exercises into their daily routine. When accessible, an online platform and ongoing monitoring would support the participants in their daily practice at home. In addition, they would assist to a weekly group session guided by experienced teachers.

Group interventions should be complemented with individual sessions considering the ex-combatants personal needs. These individual interventions may be based in previous protocols implemented in ex-combatants populations (e.g., Hermenau et al., 2013; Trujillo et al., 2017). However, new protocols should be designed grounded in the knowledge accumulated during the first stage of the proposed approach. The adherence to individual and group sessions should be closely controlled.

Given that only a few studies (Hermenau et al., 2013; Trujillo et al., 2017) have attempted to provide evidence for the effectiveness of mental health and SCAMP interventions in ex-combatants, there is urgently needed to develop a rigorous assessment of interventions effectiveness and long-term effects. Our approach proposes different measures as indicators of reintegration interventions effectiveness, these include: (a) pre-and post-intervention assessment of basic and social cognition skills, (b) pre-and post-intervention assessment of mental health symptoms, (c) pre-and post-intervention assessment of violent/aggressive behaviors and attitudes, (d) pre-and post-intervention assessment of socio-economic and occupational opportunities, and (e) self-reported acceptance of the ex-combatants by their communities and families. The use of ongoing monitoring of the intervention with principles of behavioral insights would help to promote engagement and reduce attrition.

Finally, the preparation of the receiving communities which are willing and able to accept ex-combatants is of particular importance to the success of social reintegration (Bowd and Özerdem, 2013). Reintegration processes require intervention on a societal-level. Disengagement from violence requires intervention at all levels and with all actors of the conflict, including deactivating the implicit normalization of violence within the culture. Community interventions aimed at reducing stigma and revengeful actions should be framed in new spaces of collective disarmament. Community sensitization exercises can be influential in preparing a community for reintegration and should be promoted. Education and support at this stage is vital to the development and fostering of trust between communities and ex-combatants (Bowd and Özerdem, 2013). Furthermore, the promotion of personal and collective emotional healing is needed to support the emergence of symbolic expressions as initiatives for building a national historical memory. Some successful local interventions in Colombia, such as the Mampuján weavers (Belalcazar Valencia, 2017), should be extended to other sectors of the country. These ongoing collective reintegration initiatives combine intrapersonal and collective factors toward reparation.

Post-conflict Colombia represents a unique opportunity to study the different roots of violence. The implementation of the proposed approach would contribute to the understanding of the multidimensional factors involved in violence and the efficiency of these interventions to change violent behaviors. The situated perspective supported by a comprehensive scientific assessment and the designing of evidence-based interventions constitute the major strengthens of this proposal. However, some limitations and challenges should also be acknowledged. First, the implementation of the two-stage approach critically demand long-term funding from governmental and non-governmental agencies. Second, this proposal demands a concerted effort from academics, social science and mental health professionals, as well as communities and governments. Third, as any long-term intervention, high rates of drop out or withdrawal may be present. To circumvent this issue, the adherence of ex-combatants to interventions should be closely controlled.

## Concluding Remarks

The proposed two-stage approach involves long-term interventions that critically demand funding from governmental and non-governmental agencies. The implementation of an established evidence-based intervention in post-conflict Colombia demands a concerted effort from academics, social science and mental health professionals, and communities and governments, accompanied by economic and social support. Long-term, evidence-based and established interventions would contribute to the successful reintegration and rehabilitation of individuals and communities that have been exposed to extreme violence for more than 50 years.

## Author Contributions

SB and HS-G wrote the first draft. All authors designed the proposal, searched the literature, participated in discussing the contents of the paper, contributed to editing and approved the final version of the manuscript.

## Conflict of Interest Statement

The authors declare that the research was conducted in the absence of any commercial or financial relationships that could be construed as a potential conflict of interest.
